# Time-motion analysis in men’s breaking: A longitudinal study

**DOI:** 10.1371/journal.pone.0293131

**Published:** 2023-10-19

**Authors:** Alberto Pérez-Portela, Iván Prieto-Lage, Juan Carlos Argibay-González, Xoana Reguera-López-de-la-Osa, Antonio José Silva-Pinto, Alfonso Gutiérrez-Santiago

**Affiliations:** 1 Faculty of Education and Sport, Observational Research Group, University of Vigo, Vigo, Spain; 2 Education, Physical Activity and Health Research Group, Galicia Sur Health Research Institute (IIS Galicia Sur), SERGAS-UVIGO, Vigo, Spain; Università degli Studi di Milano: Universita degli Studi di Milano, ITALY

## Abstract

Quantifying the effort of a sport confrontation by determining its temporal structure is of concern to the scientific community. In breaking this has not yet been sufficiently studied. The objectives of the study were to longitudinally analyze the temporal and sequential parameters of male breaking battles to determine the evolution of these parameters and to establish a model of temporal structure. All Red Bull BC One dancers from 2011 to 2021 (n = 152 dancers) participated. By using observational methodology, all battles were analyzed (n = 142). To obtain the results, we employed different analysis techniques: descriptive, normality tests, Student’s t-test or Mann-Whitney, one factor ANOVA or Kruskall-Wallis and effect size (Cohen’s d or Hedges’ g). The significance level established for the study was ρ ≤ 0.05. The results define the temporal and sequential structure of the battles. With these, breaking professionals will be able to develop precise and adequate training for these athletes. We conclude that approaches to dancing and battling have evolved. The effort that the athlete must exert is increasing and will therefore require better preparation to cope with the physical demands required for a sport that will be incorporated into the Olympics program in 2024.

## Introduction

All sports involve an internal technical-tactical logic that determines the effort that athletes have to make [1]. In some sports the effort is continuous (cycling, swimming, etc.) and in others the effort is intermittent (boxing, soccer, dance, etc.). In terms of the latter, there are also disciplines in which the intermittency and duration of the effort is dictated by the rules due to mandatory breaks (e.g. boxing), and others where the existing breaks are of an uncertain number and duration owing to their internal logic (e.g. judo). In breaking, effort and rest are of an indeterminate duration. While one performer intervenes (makes the effort), the other rests (pauses) before the roles are switched. The period of time in which the dancer makes effort equals the opponent’s pause time.

The pauses and actions performed by the athletes condition the type of effort to be made. In turn, this determines the most appropriate training structure and load for the athlete. Each sport requires personalized preparation, focused on covering its physical demands. Numerous authors [2] have therefore attempted to quantify these demands by determining a temporal structure, defining the distribution of effort and recovery times. These investigations, called time-motion study, have been successfully carried out in numerous sports disciplines, such as racket sports [3,4], combat sports [5], team sports [6] and individual sports [7], among others.

In spite of all this work, there are still disciplines, such as breaking, which, despite having been confirmed as an Olympic sport for the 2024 Paris games, urgently need this type of research. Moreover, as is well known, breaking is a very competitive type of dance that encompasses high-level artistic and technical movements [8], in which its athletes have become professionalized, reaching competitive levels that were unthinkable several years ago. Despite this, these athletes still do not have sufficient specific studies focused on training, psychology, physiotherapy, etc., that they can use as a guide to optimize their sports performance. Furthermore, if we take into account the fact that these athletes push their bodies to the limit using ballistic and potentially injurious movements [9], we must consider that the lack of scientific references could imply an obstacle for their professional careers. The humble origins of breaking [10] could perhaps explain this paucity of scientific literature. All that is available are studies on the improvement of physical abilities [11], the epidemiology of injuries [12], the acquisition of motor skills in dancers [13], cardiorespiratory profiles in b-boys [14] and sociological studies [15]. Despite scientific efforts made in this discipline and even with the pilot study on time-motion in breaking, for which only two world championships were analyzed [1], we still do not have longitudinal time-motion studies that allow us to analyze the evolution suffered in the sport and establish the most appropriate training loads for athletes. Therefore, to solve this problem, we have conducted a study whose objectives are to longitudinally analyze the temporal and sequential parameters of breaking battles in men (b-boys) to determine the evolution suffered in these parameters and to establish a model of temporal structure. The results of this research will determine the effort exerted by these athletes and will help coaches, sports technicians and the athletes themselves to establish an adequate and individualized training load.

## Method

### Design

Observational study to establish the temporal structure of the battles in male breaking. To do so, we used observational methodology [16]. The observational design [17] employed was nomothetic (all battles), with follow-up (the behaviors present in the breaking battles during all championships will be analyzed), and unidimensional (there was no concurrence of behaviors).

### Participants

Participants were all dancers (n = 152 dancers) who competed in 10 Red Bull BC One world championships (2011–2021). The inclusion criterion is that all championship battles had three rounds. The 2014 championship was excluded because of its competition system, which involved a changing and inconsistent number of rounds. In the 2020 championship, for reasons related to Covid-19, the participants were reduced to eight instead of 16. In total, 142 battles were analyzed. The video material was obtained secondarily (from the official Red Bull BC One YouTube channel) and the data were not generated by experimentation, which is why informed consent from the participants was not required [18]. The study was approved by the Ethics Committee of the Faculty of Education and Sport Science (University of Vigo, Application 02/0320).

### Instruments

The *ad hoc* observation instrument developed for the research consists of a series of categories that allowed the behaviors performed by the dancers during the battles to be analyzed. The instrument described in Table 1 is an exhaustive and mutually exclusive category system [16] called an Observed Temporal System for Breaking v2 (OTSB v2), this being a revision of the first version used in a pilot study [1].

**Table 1:** Observational instrument. Observed Temporal System for Breaking v2 (OTSB v2).

Category	Subcategory	Code	Description
**TOPROCK**		TR	It encompasses any movement executed while standing in order to keep up with the rhythm of the music or the beats that the song marks. It usually marks the beginning of a round.
**DOWNROCK**	**Drop**	D	Short transition that involves changing from toprock to another move (usually inside downrock). It is used to reach the floor in an original way. Usually follows the beat.
	**Footwork**	FW	Movements done on the floor using feet and hands as support. It can follow the beat directly or not and has multiple levels and approaches. It is not essential to be constantly touching the floor with an extremity. Floorrock would be one of the common levels of footwork, movements that are in direct contact with the floor, on the back, chest, shoulders, etc.
	**Spin**	S	Every spin with a minimum of 360 degrees while on the floor is considered a spin. When the breaker spins several times in the same move, it becomes part of the family of powermoves.
	**Powermove**	PM	Explosive and complex set of fine movements using centrifugal power and dynamic balance. They have more than one axis of action (sagittal, longitudinal and transversal) and often lead to a circle. All those movements of 1 to 4 support have the possibility of an aerial phase and a reception while moving. They can follow the beat or not and they can be linked in combos that we will register as several powermoves in a row.
	**BlowUp**	BU	Combinations of fast and unexpected movements that have surprise within the set of the dancer. They can contain freezes, powermoves or flips.
	**Acrobatic**	AC	It includes all movements in which there is no contact with the floor and has a well-defined aerial phase. Flips and gymnastic stunts as strict definition. It can follow the beat or not.
	**Transition**	T	The main function of these movements is the link between different categories. It depends on the imagination of the dancer and adds originality and creativity to the round.
**FREEZE**		F	Poses in which the dancer stops moving completely in the middle of a set. It provides control and pace to a dancer´s round. Inside the freezes, there can be tricks or combos that we register as several freezes in a row. They can follow the beat or not.

After designing and testing the observation instrument, construct validity was assessed by means of its consistency with the theoretical framework [19] and by consulting three experts in breaking. The experts were required to show their degree of agreement with the instrument, reaching an agreement level of 95%. The data were recorded using LINCE PLUS software [20].

### Procedure

After adequate training in the use of the instruments, the data from the battles were observed and recorded with LINCE PLUS software by two expert observers. To ensure rigor in the recording process [21], the quality of the recorded data was controlled by calculating intra- and inter-observer agreement using Cohen’s Kappa coefficient [22], calculated using LINCE PLUS software. In both cases, the calculation of the kappa coefficient was applied to all the categorical variables of the observation instrument, obtaining the mean value of all of them. Both concordances were carried out with non-matched subjects who did not belong to the final sample, in a number equivalent to one third of the final sample (n = 50). First, intra-observer agreement was performed, obtaining a mean kappa value for all categories of 0.96 in observer 1 and 0.91 in observer 2. Subsequently, inter-observer agreement was calculated, obtaining a mean kappa value for all categories of 0.90.

All battles were observed. The men’s battles comprise six rounds (three per dancer). Each athlete was studied individually, recording the movements they executed and their duration. After all the battles were recorded, we obtained an Excel file with the sequentiality and temporality of all the study behaviors. The versatility of this file allowed us to perform successive transformations for the different analyses. After obtaining the results, we made a model of the temporal structure of the battle. This model was made by consensus by a breaking expert and an expert in time-motion with ample experience in the construction of other temporal structure models [1].

### Data analysis

All of the statistical analyses were performed with the IBM Statistical Package for the Social Sciences, version 25.0 (IBM-SPSS Inc., Chicago, IL, USA). A general descriptive analysis was carried out and others were stratified according to the phase of the competition (top 16, top 8, semi-final and final), competitive periods (2011–2016 and 2017–2021) and battle rounds (1 to 6) for each of the variables under study, through measures of central tendency (mean and standard deviation). The normality of the sample was tested using the Kolmogorov-Smirnov method (with Lilliefors correction) for variables with more than 50 cases and Shapiro-Wilk for variables with 50 or fewer cases. The mean values of the sequential and temporal parameters of the battles obtained in the two competitive periods were compared by means of a Student’s t test for independent samples (when the sample was normal) and by means of the Mann-Whitney U test (when the sample was non-normal). These mean values were also compared between the different phases of the competition and between the different rounds by means of a one-factor ANOVA (applying a post hoc Tukey-b test in the case of statistically significant differences) when the sample was normal or by means of the Kruskall-Wallis test when the sample was non-normal. The significance level was considered to be p<0.05. We analyzed the effect size by Cohen’s d when the sample was normal and by Hedges’ g when the sample was non-normal, considering the following values to determine the effect size: d or g < 0.2 (null), d or g = 0.2–0.49 (small), d or g = 0.5–0.80 (moderate) and d or g > 0.8 (large).

## Results

### Analysis of the battles: Totality of the competitions, competitive periods and phases of the competition

Table 2 presents a descriptive analysis of the sequential and temporal parameters of the male battles in the totality of the competitions and in two competitive periods and a comparison between them both.

**Table 2:** Descriptive analysis (total and by competitive periods), comparison of the competitive periods and size of effect of the sequential and temporal parameters of the breaking battles.

Study variables	Nor	2011–2021					2011–2016			2017–2021			Comparison		Cohen’s d or Hedges’ g
		n	Mean	SD	95% CI		n	Mean	SD	n	Mean	SD	t or U	p	
					Low	Up									d or g
***GTP***															
Battle Time	No	142	182.27	40.13	175.61	188.93	75	172.31	44.30	67	193.42	31.65	1537.500	0.000*	-0.540
Toprock Time	No	142	53.14	20.38	49.76	56.52	75	48.52	20.17	67	58.31	19.49	1651.000	0.000*	-0.491
Drop Time	No	142	7.06	3.39	6.49	7.62	75	6.17	3.59	67	8.04	2.87	1569.000	0.000*	-0.569
Footwork Time	No	142	57.63	20.66	54.21	61.06	75	50.73	16.36	67	65.36	22.29	1479.000	0.000*	-0.750
Spin Time	No	123	5.08	4.06	4.36	5.81	64	4.77	3.94	59	5.42	4.20	1678.000	0.284	
Powermove Time	No	142	20.82	11.22	18.96	22.69	75	22.25	11.66	67	19.22	10.57	2116.500	0.105	
BlowUp Time	No	121	7.73	5.95	6.66	8.80	57	7.26	6.62	64	8.14	5.31	1559.000	0.167	
Acrobatic Time	No	78	3.01	2.35	2.48	3.54	43	3.14	2.31	35	2.86	2.44	671.000	0.400	
Transition Time	Yes	142	14.04	5.97	13.04	15.03	75	15.31	5.79	67	12.61	5.90	2.746	0.007*	0.462
Freeze Time	No	142	17.06	7.53	15.81	18.31	75	17.99	8.43	67	16.03	6.28	2231.000	0.249	
***GSP***															
# of elements	Yes	142	77.77	15.59	75.19	80.36	75	76.80	17.05	67	78.87	13.83	-0.787	0.433	
# of Toprock	No	142	8.35	2.58	7.92	8.78	75	7.37	2.20	67	9.45	2.55	1303.500	0.000*	-0.870
# of Drop	No	142	7.56	3.25	7.02	8.10	75	6.83	3.31	67	8.37	3.00	1706.500	0.001*	-0.485
# of Footwork	No	142	16.03	4.94	15.21	16.85	75	14.85	4.60	67	17.34	5.02	1707.500	0.001*	-0.516
# of Spin	No	123	3.43	2.37	3.01	3.85	64	3.31	2.58	59	3.56	2.14	1613.500	0.158	
# of Powermove	No	142	15.57	8.14	14.22	16.92	75	16.47	8.22	67	14.57	7.99	2161.500	0.151	
# of BlowUp	No	121	2.41	1.57	2.13	2.70	57	2.02	1.48	64	2.77	1.58	1320.000	0.006*	-0.484
# of Acrobatic	No	78	1.97	1.49	1.64	2.31	43	2.09	1.51	35	1.83	1.48	674.500	0.384	
# of Transition	Yes	142	10.89	4.11	10.21	11.58	75	11.59	3.81	67	10.12	4.31	2.152	0.033*	0.362
# of Freeze	No	142	13.26	5.42	12.36	14.16	75	14.13	6.35	67	12.28	3.98	2171.500	0.163	
***PTP***															
Element Time	No	142	2.35	0.53	2.26	2.43	75	2.23	0.42	67	2.48	0.61	1998.000	0.009*	-0.480
Toprock Time	No	141	6.47	1.93	6.15	6.79	75	6.49	1.69	66	6.44	2.18	2350.000	0.601	
Drop Time	No	142	1.01	0.12	0.99	1.03	75	1.00	0.00	67	1.03	0.17	2437.500	0.133	
Footwork Time	No	142	3.67	0.96	3.51	3.83	75	3.52	0.81	67	3.84	1.08	2154.000	0.117	
Spin Time	No	123	1.53	0.92	1.36	1.69	64	1.53	0.89	59	1.53	0.95	1837.000	0.758	
Powermove Time	No	142	1.25	0.43	1.17	1.32	75	1.29	0.46	67	1.19	0.40	2263.000	0.172	
BlowUp Time	No	121	3.26	2.16	2.88	3.65	57	3.54	2.62	64	3.02	1.64	1596.500	0.223	
Acrobatic Time	No	78	1.55	0.91	1.35	1.76	43	1.56	0.88	35	1.54	0.95	733.000	0.818	
Transition Time	No	142	1.19	0.39	1.12	1.26	75	1.23	0.42	67	1.15	0.36	2318.000	0.242	
Freeze Time	No	142	1.22	0.41	1.15	1.29	75	1.21	0.41	67	1.22	0.42	2486.000	0.880	

* *p < 0*.*05*.

Nor = Normality; CI = Confidence interval; GTP = Global Temporal Parameters; GSP = *Global* Sequential Parameters; PTP = Partial Temporal Parameters.

Significant differences (effect size in parentheses) were observed between the two competitive periods in several global temporal parameters. The values are higher in the second competitive period for battle time (moderate), toprock time (small), drop time (moderate) and footwork time (moderate). And higher in the first competitive period in terms of transition time (small). Regarding the sequential parameters, we see greater use of toprocks (large), drops (small), footwork (moderate) and blowups (small) in the latter years and a higher use of transitions (small) in the former. Furthermore, the average time of an element had a small increase in the second competitive period.

Table 3 shows an analysis of the battles according to the phases of the competition (top 16, top 8, semi-final and final). The only differences are in terms of the total number of footwork elements.

**Table 3:** Descriptive analysis of the different phases of the competition and comparison among them (ANOVA or Kruskal-Wallis) of the sequential and temporal parameters of the breaking battles.

Study variables	Nor	TOP16			TOP8			SF			FINAL			ANOVA or Kruskal-Wallis		
		n	Mean	SD	n	Mean	SD	n	Mean	SD	n	Mean	SD	F or H	gl	Sig.
***GTP***																
Battle Time	No	72	180.65	42.36	40	179.43	37.38	20	181.05	27.78	10	207.70	51.14	2.360	3	0.501
Toprock Time	No	72	53.42	21.72	40	54.08	18.61	20	49.35	17.98	10	55.00	23.73	0.391	3	0.942
Drop Time	No	72	6.85	3.07	40	6.53	3.15	20	7.60	3.28	10	9.60	5.56	3.718	3	0.294
Footwork Time	No	72	57.06	22.39	40	54.30	18.28	20	59.25	17.03	10	71.90	19.67	7.553	3	0.056
Spin Time	No	63	4.56	3.55	35	5.23	4.51	17	5.65	3.87	8	7.38	5.78	2.418	3	0.490
Powermove Time	No	72	20.44	11.31	40	21.55	10.94	20	20.05	12.12	10	22.20	11.28	1.193	3	0.755
BlowUp Time	No	62	8.26	6.81	33	7.12	4.44	17	7.71	5.70	9	6.33	5.27	0.774	3	0.856
AcrobaticTime	No	42	3.29	2.55	18	3.00	2.54	16	2.38	1.63	2	2.50	0.71	0.920	3	0.821
Transition Time	Yes	72	14.19	5.96	40	13.98	6.17	20	13.50	6.11	10	14.20	5.79	0.073	3	0.974
Freeze Time	No	72	15.93	6.72	40	17.08	7.56	20	18.05	8.17	10	23.20	9.46	7.149	3	0.067
***GSP***																
# of elements	Yes	72	77.38	14.67	40	74.70	16.06	20	80.00	13.19	10	88.50	21.08	2.310	3	0.079
# of Toprock	No	72	8.28	2.60	40	8.05	2.61	20	8.80	2.65	10	9.20	2.25	4.083	3	0.253
# of Drops	No	72	7.44	3.15	40	6.83	2.99	20	8.30	2.81	10	9.80	4.76	5.548	3	0.136
# of Footwork	No	72	16.10	5.19	40	14.63	3.82	20	16.75	4.19	10	19.70	6.72	9.515	3	0.023*
# of Spin	No	63	3.24	2.08	35	3.20	2.48	17	4.35	2.96	8	4.00	2.62	2.749	3	0.432
# of Powermove	No	72	15.56	8.55	40	15.83	7.80	20	14.80	7.71	10	16.20	8.40	0.659	3	0.883
# of BlowUp	No	62	2.56	1.72	33	2.18	1.42	17	2.47	1.42	9	2.11	1.36	1.482	3	0.686
# of Acrobatic	No	42	2.17	1.71	18	2.00	1.28	16	1.50	1.10	2	1.50	0.71	3.096	3	0.377
# of Transition	Yes	72	10.96	4.15	40	10.88	4.13	20	10.65	4.30	10	11.00	3.94	0.031	3	0.993
# of Freeze	No	72	12.74	4.88	40	13.00	6.19	20	13.70	4.77	10	17.20	6.27	6.372	3	0.095
***PTP***																
Element Time	No	72	2.36	0.51	40	2.43	0.59	20	2.20	0.52	10	2.20	0.42	3.798	3	0.284
Toprock Time	No	72	6.53	2.04	40	6.83	1.89	19	5.68	1.53	10	6.10	1.66	4.298	3	0.231
Drop Time	No	72	1.00	0.00	40	1.05	0.22	20	1.00	0.00	10	1.00	0.00	5.136	3	0.162
Footwork Time	No	72	3.61	1.03	40	3.78	1.00	20	3.60	0.75	10	3.80	0.63	2.184	3	0.535
Spin Time	No	63	1.48	0.91	35	1.71	1.07	17	1.24	0.56	8	1.75	0.71	5.905	3	0.116
Powermove Time	No	72	1.22	0.42	40	1.25	0.44	20	1.25	0.44	10	1.40	0.52	1.491	3	0.684
BlowUp Time	No	62	3.10	1.43	33	3.55	2.02	17	3.65	4.21	9	2.67	1.22	2.093	3	0.553
Acrobatic Time	No	42	1.57	0.94	18	1.33	0.59	16	1.69	1.08	2	2.00	1.41	1.503	3	0.682
Transition Time	No	72	1.21	0.41	40	1.20	0.41	20	1.15	0.37	10	1.10	0.32	0.910	3	0.823
Freeze Time	No	72	1.17	0.38	40	1.33	0.47	20	1.10	0.31	10	1.40	0.52	7.316	3	0.062

* *p < 0*.*05*.

Nor = Normality; GTP = Global Temporal Parameters; GSP = Global Sequential Parameters; PTP = Partial Temporal Parameters.

### Analysis of battle rounds: Overall championships (2011–2021), first competitive period (2011–2016), second competitive period (2017–2021) and comparison between competitive periods (2011–2016 vs 2017–2021)

Table 4 presents a description of the temporal and sequential parameters of each of the battle rounds and a comparison between them in the totality of the championships. We found significant differences in the total round times, toprocks, drops and powermoves, in the total number of elements per round, in the total number of drops, powermoves, acrobatic elements and transitions, and in the average time of an element and a toprock. It should be noted that each dancer does not appear in all six rounds but in three, alternating between question (first dancer) and answer (second dancer).

**Table 4:** Descriptive analysis of the rounds (2011–2021) and comparison between rounds (Kruskal-Wallis) of the sequential and temporal parameters of the breaking battles.

Study variables	Nor	Round1			Round2			Round3			Round4			Round5			Round6			Kruskal-Wallis		
		n	Mean	SD	n	Mean	SD	n	Mean	SD	n	Mean	SD	n	Mean	SD	n	Mean	SD	H	gl	Sig.
***GTP***																						
Round Time	No	142	34.35	9.74	142	33.10	7.63	142	29.24	8.23	142	27.93	6.56	130	30.58	9.39	130	28.42	5.94	66.432	5	0.000*
Toprock Time	No	141	11.05	5.45	137	10.23	4.63	129	8.74	4.96	130	7.93	4.40	125	10.14	6.05	126	7.91	4.33	48.943	5	0.000*
Drop Time	No	121	1.66	1.00	109	1.70	0.89	117	1.56	0.87	111	1.33	0.69	114	1.54	0.85	100	1.60	0.91	14.673	5	0.012*
Footwork Time	No	140	10.16	5.55	133	9.96	4.91	140	9.75	4.99	137	9.66	4.69	126	10.47	5.18	123	10.37	4.77	3.497	5	0.624
Spin Time	No	49	2.20	1.76	68	2.26	1.80	46	1.80	1.24	50	1.98	1.39	32	2.03	1.23	41	2.49	2.03	4.518	5	0.477
Powermove Time	No	114	5.12	3.09	122	5.36	3.43	107	4.21	2.73	113	3.95	2.98	92	4.02	2.79	92	4.33	3.24	21.771	5	0.001*
BlowUp Time	No	37	4.19	3.35	38	3.26	1.72	53	3.15	2.12	48	4.04	2.63	35	3.74	1.84	39	3.54	2.50	5.536	5	0.354
Acrobatic Time	No	22	1.95	1.25	28	2.21	1.60	20	1.60	1.19	21	2.33	1.68	17	1.35	0.70	14	1.57	0.76	7.651	5	0.177
Transition Time	No	128	3.11	1.98	128	2.88	1.78	126	2.96	1.94	114	2.68	1.71	105	2.95	1.91	96	2.44	1.79	10.469	5	0.063
Freeze Time	No	131	3.20	1.78	123	3.68	2.78	130	3.04	2.01	128	3.10	2.09	112	3.03	2.17	115	3.39	2.11	6.211	5	0.286
***GSP***																						
# of elements	No	142	14.49	4.45	142	14.70	4.14	142	12.90	3.43	142	11.99	3.39	130	12.12	4.26	130	11.99	3.63	64.892	5	0.000*
# of Toprock	No	141	1.55	0.81	137	1.53	0.85	130	1.42	0.74	130	1.40	0.69	125	1.61	1.11	126	1.43	0.61	6.975	5	0.223
# of Drop	No	121	1.66	0.89	109	1.72	0.84	117	1.59	0.78	110	1.35	0.58	114	1.52	0.73	100	1.51	0.66	13.569	5	0.019*
# of Footwork	No	140	2.86	1.28	133	2.80	1.27	140	2.73	1.15	137	2.69	1.08	126	2.74	1.15	123	2.90	1.31	2.153	5	0.828
# of Spin	No	49	1.43	0.79	68	1.66	1.22	46	1.39	0.65	50	1.22	0.71	32	1.34	0.70	41	1.49	0.93	9.336	5	0.096
# of Powermove	No	114	3.91	2.35	122	4.08	2.72	107	3.20	2.03	113	2.84	2.01	92	2.86	1.94	92	3.14	2.09	29.276	5	0.000*
# of BlowUp	No	37	1.27	0.51	38	1.18	0.39	53	1.08	0.27	48	1.10	0.31	35	1.11	0.32	39	1.21	0.47	6.916	5	0.227
# of Acrobatic	No	22	1.27	0.77	28	1.54	0.92	20	1.10	0.45	21	1.29	0.90	17	1.00	0.00	14	1.07	0.27	12.572	5	0.028*
# of Transition	No	128	2.43	1.23	128	2.23	1.10	126	2.19	1.14	113	2.04	1.09	105	2.23	1.22	96	1.91	1.03	14.715	5	0.012*
# of Freeze	No	131	2.57	1.31	123	2.72	1.74	130	2.45	1.53	128	2.42	1.58	112	2.34	1.43	115	2.40	1.34	6.900	5	0.228
***PTP***																						
Element Time	No	142	2.54	1.02	142	2.41	0.72	142	2.39	0.93	142	2.47	0.89	130	2.75	1.00	130	2.58	1.03	12.742	5	0.026*
Toprock Time	No	140	8.02	4.31	137	7.74	4.00	130	6.89	4.39	130	6.20	3.42	125	7.23	4.24	124	6.07	3.52	26.630	5	0.000*
Drop Time	No	121	1.13	0.36	109	1.11	0.34	117	1.10	0.40	110	1.04	0.19	114	1.10	0.38	100	1.17	0.62	6.740	5	0.241
Footwork Time	No	140	3.99	2.45	133	3.89	2.22	140	3.96	2.62	137	3.92	2.06	126	4.34	2.51	123	3.87	1.93	3.761	5	0.584
Spin Time	No	49	1.59	1.32	68	1.46	1.18	46	1.35	0.71	50	1.74	1.23	32	1.56	0.84	41	1.80	1.47	10.466	5	0.063
Powermove Time	No	114	1.36	0.61	122	1.37	0.78	107	1.36	0.76	113	1.50	0.97	92	1.57	1.21	92	1.46	1.01	3.622	5	0.605
BlowUp Time	No	37	3.46	3.25	38	2.79	1.32	53	2.96	2.06	48	3.77	2.60	35	3.49	1.80	39	3.03	2.12	5.696	5	0.337
Acrobatic Time	No	22	1.59	1.05	28	1.50	0.84	20	1.55	1.19	21	1.90	1.14	17	1.35	0.70	14	1.50	0.76	4.150	5	0.528
Transition Time	No	128	1.30	0.60	128	1.27	0.53	126	1.37	0.69	113	1.35	0.58	105	1.33	0.58	96	1.31	1.08	3.333	5	0.649
Freeze Time	No	131	1.28	0.54	123	1.37	0.58	130	1.30	0.51	128	1.36	0.61	112	1.39	0.79	115	1.55	1.15	4.374	5	0.497

* *p < 0*.*05*.

Nor = Normality; GTP = Global Temporal Parameters; GSP = Global Sequential Parameters; PTP = Partial Temporal Parameters.

Table 5 describes the temporal and sequential parameters of each of the battle rounds in the first competitive period, comparing them with one another. We found significant differences in the total round times, toprocks and powermoves, in the total number of elements per round, in the total number of spins and powermoves and in the average time of an element and a toprock.

**Table 5:** Descriptive analysis of the rounds of the 2011–2016 competitive period and comparison between rounds (Kruskal-Wallis) of the sequential and temporal parameters of the breaking battles.

Study variables	Nor	Round1			Round2			Round3			Round4			Round5			Round6			Kruskal-Wallis		
		n	Mean	SD	n	Mean	SD	n	Mean	SD	n	Mean	SD	n	Mean	SD	n	Mean	SD	H	gl	Sig.
**GTP**																						
Round Time	No	75	32.33	9.69	75	32.48	8.02	75	27.36	7.29	75	26.96	6.77	63	27.97	8.52	63	27.06	5.38	35.448	5	0.000*
Toprock Time	No	74	10.82	5.76	72	9.83	4.40	66	7.94	4.73	67	7.21	3.86	59	9.19	5.58	60	7.00	3.42	35.498	5	0.000*
Drop Time	No	65	1.58	1.07	53	1.53	0.85	65	1.46	0.87	53	1.19	0.52	54	1.41	0.66	46	1.50	0.94	8.174	5	0.147
Footwork Time	No	74	8.99	4.52	69	8.90	4.27	74	8.19	4.31	71	8.49	4.14	61	9.87	4.78	59	9.53	4.34	7.592	5	0.180
Spin Time	No	19	2.11	1.41	38	2.53	1.83	24	1.88	1.39	30	1.90	1.37	12	2.00	1.35	11	2.73	1.27	6.927	5	0.226
Powermove Time	No	60	5.17	3.08	66	5.68	3.69	59	4.17	2.65	64	4.33	3.46	46	3.72	2.75	46	4.91	3.78	13.343	5	0.020*
BlowUp Time	No	12	5.58	4.72	17	3.12	1.50	24	3.54	2.69	23	4.00	2.43	9	3.78	1.99	16	3.88	3.12	4.286	5	0.509
Acrobatic Time	No	12	1.83	1.03	16	2.50	1.83	10	1.40	0.70	14	2.50	1.83	9	1.11	0.33	8	1.38	0.52	9.608	5	0.087
Transition Time	No	67	3.22	2.07	70	3.14	2.03	67	3.31	2.02	63	2.95	1.73	52	3.04	1.57	51	2.57	1.69	5.640	5	0.343
Freeze Time	No	68	3.06	1.44	65	4.12	3.27	72	3.06	2.03	70	3.41	2.32	52	3.08	2.01	60	3.52	2.16	3.904	5	0.563
**GSP**																						
# of elements	No	75	13.55	3.69	75	14.84	4.66	75	12.93	3.09	75	12.37	3.01	63	11.60	4.03	63	12.27	3.48	26.383	5	0.000*
# of Toprock	No	74	1.38	0.61	72	1.31	0.52	67	1.37	0.69	67	1.31	0.66	59	1.51	1.37	60	1.30	0.50	1.761	5	0.881
# of Drop	No	65	1.49	0.75	53	1.53	0.85	65	1.51	0.79	52	1.27	0.49	54	1.44	0.66	46	1.43	0.72	3.259	5	0.660
# of Footwork	No	74	2.64	1.20	69	2.61	1.25	74	2.58	1.11	71	2.54	0.91	61	2.62	1.07	59	2.68	1.27	0.372	5	0.996
# of Spin	No	19	1.32	0.75	38	1.87	1.47	24	1.33	0.64	30	1.20	0.66	12	1.17	0.39	11	2.18	1.33	16.188	5	0.006*
# of Powermove	No	60	3.88	2.16	66	4.33	3.08	59	3.07	1.82	64	3.00	2.20	46	2.72	1.93	46	3.61	2.38	16.912	5	0.005*
# of BlowUp	No	12	1.17	0.39	17	1.12	0.33	24	1.04	0.20	23	1.13	0.34	9	1.00	0.00	16	1.13	0.34	2.902	5	0.715
# of Acrobatic	No	12	1.33	0.89	16	1.63	1.09	10	1.20	0.63	14	1.21	0.80	9	1.00	0.00	8	1.00	0.00	7.511	5	0.185
# of Transition	No	67	2.42	1.22	70	2.36	1.19	67	2.37	1.11	62	2.18	1.00	52	2.19	1.14	51	2.08	1.02	3.820	5	0.576
# of Freeze	No	68	2.53	1.28	65	2.94	2.02	72	2.50	1.56	70	2.69	1.63	52	2.58	1.49	60	2.48	1.38	1.709	5	0.888
**PTP**																						
Element Time	No	75	2.49	0.94	75	2.37	0.71	75	2.19	0.73	75	2.21	0.58	63	2.67	1.05	63	2.35	0.77	12.293	5	0.031*
Toprock Time	No	74	8.42	4.19	72	8.19	3.90	67	6.37	3.96	67	5.91	3.00	59	7.10	3.74	60	5.77	2.88	32.367	5	0.000*
Drop Time	No	65	1.17	0.42	53	1.13	0.39	65	1.08	0.32	52	1.02	0.14	54	1.06	0.23	46	1.11	0.38	8.521	5	0.130
Footwork Time	No	74	3.73	1.92	69	3.90	2.62	74	3.45	1.88	71	3.63	1.77	61	4.39	2.87	59	3.93	1.99	4.536	5	0.475
Spin Time	No	19	1.79	1.36	38	1.45	0.86	24	1.46	0.83	30	1.70	1.26	12	1.67	0.78	11	1.36	0.50	2.365	5	0.797
Powermove Time	No	60	1.33	0.57	66	1.44	0.86	59	1.42	0.86	64	1.48	0.69	46	1.52	1.03	46	1.37	0.74	3.001	5	0.700
BlowUp Time	No	12	5.08	4.81	17	2.88	1.32	24	3.42	2.64	23	3.61	2.33	9	3.78	1.99	16	3.50	2.99	3.255	5	0.661
Acrobatic Time	No	12	1.42	0.67	16	1.63	1.02	10	1.30	0.67	14	2.14	1.29	9	1.11	0.33	8	1.38	0.52	7.702	5	0.173
Transition Time	No	67	1.36	0.62	70	1.30	0.57	67	1.43	0.61	62	1.35	0.63	52	1.44	0.67	51	1.20	0.40	6.389	5	0.270
Freeze Time	No	68	1.32	0.58	65	1.43	0.66	72	1.26	0.47	70	1.33	0.63	52	1.29	0.54	60	1.50	0.72	5.404	5	0.369

* *p < 0*.*05*.

Nor = Normality; GTP = Global Temporal Parameters; GSP = Global Sequential Parameters; PTP = Partial Temporal Parameters.

In Table 6 we analyze the second competitive period. There are significant differences between the rounds in total round time and toprock and powermove time, in the total number of elements per round, the total number of drops, powermoves and transitions and the average time of a spin.

**Table 6:** Descriptive analysis of the rounds of the 2017–2021 competitive period and comparison between rounds (Kruskal-Wallis) of the sequential and temporal parameters of the breaking battles.

Study variables	Nor	Round1			Round2			Round3			Round4			Round5			Round6			Kruskal-Wallis		
		n	Mean	SD	n	Mean	SD	n	Mean	SD	n	Mean	SD	n	Mean	SD	n	Mean	SD	H	gl	Sig.
***GTP***																						
Round Time	No	67	36.60	9.36	67	33.79	7.17	67	31.34	8.76	67	29.01	6.19	67	33.03	9.56	67	29.69	6.20	40.857	5	0.000*
Toprock Time	No	67	11.30	5.13	65	10.68	4.86	63	9.59	5.09	63	8.70	4.82	66	11.00	6.36	66	8.74	4.90	16.758	5	0.005*
Drop Time	No	56	1.75	0.90	56	1.86	0.90	52	1.69	0.88	58	1.47	0.80	60	1.65	0.99	54	1.69	0.89	8.484	5	0.131
Footwork Time	No	66	11.47	6.29	64	11.11	5.32	66	11.50	5.15	66	10.92	4.95	65	11.03	5.51	64	11.16	5.05	1.056	5	0.958
Spin Time	No	30	2.27	1.96	30	1.93	1.74	22	1.73	1.08	20	2.10	1.45	20	2.05	1.19	30	2.40	2.25	2.445	5	0.785
Powermove Time	No	54	5.07	3.12	56	4.98	3.08	48	4.25	2.86	49	3.45	2.15	46	4.33	2.82	46	3.74	2.51	12.498	5	0.029*
BlowUp Time	No	25	3.52	2.28	21	3.38	1.91	29	2.83	1.47	25	4.08	2.84	26	3.73	1.82	23	3.30	2.01	3.976	5	0.553
Acrobatic Time	No	10	2.10	1.52	12	1.83	1.19	10	1.80	1.55	7	2.00	1.41	8	1.63	0.92	6	1.83	0.98	0.619	5	0.987
Transition Time	No	61	2.98	1.89	58	2.55	1.37	59	2.56	1.78	51	2.35	1.65	53	2.87	2.21	45	2.29	1.90	7.674	5	0.175
Freeze Time	No	63	3.35	2.08	58	3.19	2.02	58	3.02	2.00	58	2.72	1.71	60	2.98	2.31	55	3.25	2.07	4.366	5	0.498
***GSP***																						
# of elements	No	67	15.55	4.99	67	14.55	3.50	67	12.87	3.81	67	11.55	3.73	67	12.61	4.44	67	11.73	3.77	46.042	5	0.000*
# of Toprock	No	67	1.73	0.95	65	1.78	1.05	63	1.48	0.78	63	1.49	0.72	66	1.70	0.82	66	1.55	0.68	7.530	5	0.184
# of Drop	No	56	1.86	1.00	56	1.89	0.80	52	1.69	0.76	58	1.43	0.65	60	1.58	0.79	54	1.57	0.60	14.002	5	0.016*
# of Footwork	No	66	3.11	1.34	64	3.02	1.27	66	2.89	1.18	66	2.86	1.23	65	2.85	1.23	64	3.11	1.32	2.587	5	0.763
# of Spin	No	30	1.50	0.82	30	1.40	0.72	22	1.45	0.67	20	1.25	0.79	20	1.45	0.83	30	1.23	0.57	5.418	5	0.367
# of Powermove	No	54	3.94	2.57	56	3.79	2.22	48	3.35	2.26	49	2.63	1.74	46	3.00	1.97	46	2.67	1.65	16.200	5	0.006*
# of BlowUp	No	25	1.32	0.56	21	1.24	0.44	29	1.10	0.31	25	1.08	0.28	26	1.15	0.37	23	1.26	0.54	5.627	5	0.344
# of Acrobatic	No	10	1.20	0.63	12	1.42	0.67	10	1.00	0.00	7	1.43	1.13	8	1.00	0.00	6	1.17	0.41	6.568	5	0.255
# of Transition	No	61	2.44	1.25	58	2.07	0.97	59	1.98	1.14	51	1.86	1.18	53	2.26	1.30	45	1.71	1.01	16.494	5	0.006*
# of Freeze	No	63	2.62	1.35	58	2.47	1.33	58	2.38	1.51	58	2.10	1.47	60	2.13	1.36	55	2.31	1.29	10.283	5	0.068
***PTP***																						
Element Time	No	67	2.58	1.10	67	2.45	0.72	67	2.63	1.07	67	2.76	1.07	67	2.82	0.95	67	2.81	1.18	9.274	5	0.099
Toprock Time	No	66	7.58	4.42	65	7.23	4.07	63	7.44	4.78	63	6.51	3.81	66	7.35	4.67	64	6.36	4.03	4.569	5	0.471
Drop Time	No	56	1.09	0.29	56	1.09	0.29	52	1.13	0.49	58	1.05	0.22	60	1.13	0.47	54	1.22	0.77	2.238	5	0.815
Footwork Time	No	66	4.29	2.91	64	3.88	1.70	66	4.53	3.18	66	4.23	2.30	65	4.29	2.13	64	3.81	1.89	3.150	5	0.677
Spin Time	No	30	1.47	1.31	30	1.47	1.50	22	1.23	0.53	20	1.80	1.20	20	1.50	0.89	30	1.97	1.67	14.061	5	0.015*
Powermove Time	No	54	1.39	0.66	56	1.29	0.68	48	1.29	0.62	49	1.51	1.26	46	1.61	1.37	46	1.54	1.22	3.104	5	0.684
BlowUp Time	No	25	2.68	1.80	21	2.71	1.35	29	2.59	1.35	25	3.92	2.87	26	3.38	1.77	23	2.70	1.18	6.822	5	0.234
Acrobatic Time	No	10	1.80	1.40	12	1.33	0.49	10	1.80	1.55	7	1.43	0.53	8	1.63	0.92	6	1.67	1.03	0.393	5	0.996
Transition Time	No	61	1.25	0.57	58	1.24	0.47	59	1.29	0.77	51	1.33	0.52	53	1.23	0.47	45	1.44	1.52	2.581	5	0.764
Freeze Time	No	63	1.24	0.50	58	1.29	0.46	58	1.34	0.55	58	1.40	0.59	60	1.48	0.95	55	1.60	1.49	3.761	5	0.584

* *p < 0*.*05*.

Nor = Normality; GTP = Global Temporal Parameters; GSP = Global Sequential Parameters; PTP = Partial Temporal Parameters.

In Table 7 we show a comparison of the rounds between both competitive periods in order to examine their evolution in terms of competing. Differences were found in the following categories: total time of rounds, toprocks, drops, footworks, spins, transitions and freezes and the average time of an element, footwork, a blowup and a transition.

**Table 7:** Comparison of rounds between competitive periods (2011–2016 vs 2017–2021) of the sequential and temporal parameters of the breaking battles.

Study variables	Nor	Round1			Round2			Round3			Round4			Round5			Round6		
		U Mann-Whitney		H-g	U Mann-Whitney		H-g	U Mann-Whitney		H-g	U Mann-Whitney		H-g	U Mann-Whitney		H-g	U Mann-Whitney		H-g
		U	p	g	U	p	g	U	p	g	U	p	g	U	p	g	U	p	g
***GTP***																			
Round Time	No	1787.500	0.003*	-0.445	2161.000	0.150		1745.000	0.002*	-0.494	2007.500	0.039*	-0.314	1328.500	0.000*	-0.555	1609.500	0.019*	-0.448
Toprock Time	No	2261.500	0.368		2124.000	0.350		1647.000	0.041*	-0.334	1667.000	0.038*	-0.340	1622.500	0.108		1611.000	0.070	
Drop Time	No	1513.000	0.071		1151.000	0.026*	-0.373	1382.000	0.049*	-0.263	1242.500	0.019*	-0.404	1441.000	0.237		1009.500	0.067	
Footwork Time	No	1888.000	0.020*	-0.455	1694.500	0.020*	-0.457	1399.500	0.000*	-0.697	1622.000	0.002*	-0.532	1759.500	0.275		1515.500	0.059	
Spin Time	No	270.000	0.737		438.500	0.079		263.500	0.990		284.000	0.728		116.000	0.867		113.000	0.111	
Powermove Time	No	1571.000	0.779		1692.500	0.422		1405.000	0.945		1392.000	0.302		917.000	0.266		896.500	0.203	
BlowUp Time	No	103.500	0.126		169.500	0.787		311.000	0.501		278.000	0.842		115.500	0.954		180.000	0.907	
Acrobatic Time	No	58.500	0.914		80.000	0.429		45.000	0.654		42.500	0.610		25.500	0.174		18.000	0.384	
Transition Time	No	1934.500	0.596		1753.500	0.176		1475.500	0.012*	0.391	1220.500	0.024*	0.352	1180.500	0.196		983.500	0.210	
Freeze Time	No	2053.500	0.678		1687.000	0.309		2080.500	0.971		1685.000	0.092		1434.500	0.454		1532.500	0.505	
***GSP***																			
# of elements	No	1854.500	0.007*	-0.459	2511.500	0.997		2459.000	0.826		2105.500	0.095		1818.000	0.171		1914.000	0.358	
# of Toprock	No	1919.000	0.008*	-0.445	1746.000	0.003*	-0.583	1967.500	0.414		1794.500	0.067		1531.000	0.019*	-0.168	1626.000	0.041*	-0.405
# of Drop	No	1429.000	0.024*	-0.414	1061.000	0.005*	-0.440	1412.500	0.087		1342.500	0.216		1495.500	0.416		1038.000	0.107	
# of Footwork	No	1966.000	0.041*	-0.370	1799.500	0.058		2074.500	0.111		2006.500	0.132		1795.000	0.342		1529.000	0.062	
# of Spin	No	248.500	0.345		490.000	0.245		236.000	0.446		299.000	0.970		102.000	0.354		85.000	0.003*	1.118
# of Powermove	No	1561.000	0.734		1792.500	0.773		1357.000	0.706		1449.500	0.481		951.500	0.394		843.000	0.086	
# of BlowUp	No	132.000	0.434		157.000	0.347		326.500	0.401		273.000	0.572		99.000	0.218		166.000	0.441	
# of Acrobatic	No	56.000	0.659		93.500	0.888		45.000	0.317		45.500	0.608		36.000	1.000		20.000	0.248	
# of Transition	No	2015.500	0.890		1797.000	0.243		1525.500	0.021*	0.345	1202.000	0.019*	0.288	1373.500	0.976		878.500	0.035*	0.359
# of Freeze	No	2072.500	0.741		1699.500	0.333		1968.500	0.562		1523.000	0.012*	0.371	1239.000	0.051		1535.500	0.507	
***PTP***																			
Element Time	No	2494.000	0.932		2392.000	0.581		1977.000	0.016*	-0.483	1759.000	0.001*	-0.642	1924.000	0.351		1577.000	0.007*	-0.453
Toprock Time	No	2074.500	0.123		1962.500	0.103		1835.500	0.198		2003.000	0.614		1911.500	0.860		1880.500	0.842	
Drop Time	No	1700.000	0.275		1446.000	0.659		1631.500	0.487		1459.000	0.366		1546.500	0.372		1188.000	0.491	
Footwork Time	No	2363.500	0.739		1964.500	0.262		1896.500	0.021*	-0.418	2013.000	0.148		1882.500	0.620		1850.000	0.844	
Spin Time	No	235.000	0.186		519.500	0.410		232.000	0.345		287.500	0.778		100.000	0.367		121.500	0.159	
Powermove Time	No	1567.500	0.709		1717.500	0.373		1326.500	0.465		1393.000	0.222		1046.500	0.914		1007.500	0.617	
BlowUp Time	No	85.000	0.030*	0.763	169.000	0.770		294.500	0.329		284.000	0.941		103.000	0.587		170.000	0.677	
Acrobatic Time	No	57.000	0.810		88.000	0.660		40.500	0.373		34.500	0.245		25.500	0.174		22.000	0.762	
Transition Time	No	1838.000	0.194		1974.500	0.719		1644.000	0.041*	0.209	1559.500	0.877		1168.500	0.086		1087.000	0.536	
Freeze Time	No	2009.000	0.408		1736.500	0.357		1953.000	0.417		1875.500	0.357		1416.500	0.294		1535.000	0.438	

* *p < 0*.*05*.

Nor = Normality; H-g = Hedges’ g; GTP = Global Temporal Parameters; GSP = Global Sequential Parameters; PTP = Partial Temporal Parameters.

## Discussion

### Discussion of the results

Regarding the results of this study, we would like to highlight that the average time of a battle is 182 s. Battles are short but concentrate a great deal of effort and wear and tear in short periods of time, similar to climbing, gymnastics or certain martial arts [1,14,23]. Battle time is superior in the second competitive period. The difference between 172 s and 193 s may seem irrelevant, but in a confrontation it can mean more information and greater intensity or cardiovascular wear and tear that can have a negative impact on following rounds and make the difference between victory and defeat [1,14,24]. This change, together with the increase in toprocks, drops and footwork, both temporarily and sequentially, indicates that breaking is focusing more on elements that bring aesthetics and content to a battle, without seeking an insubstantial increase in its duration.

We consider that the categories that have not presented a noticeable change are also important, since their neutrality indicates the relevance and permanence of the physical pillar, as is the case of the powermove [1].

Therefore, our results confirm the stability of the physical part of breaking, especially the powermove [1], and the importance attached to aesthetics and fluidity, which concurs with the judging system to be used in the Olympics, the Trivium [8,10,25–27].

The analysis of the battles according to the competitive phase shows that footwork is more prevalent in the final, as is the case with most categories. This is because the final concentrates the most information (and sometimes the most powerful information). Footwork is most likely a type of wild card used to highlight the final movements of the artist. It comes as no surprise that this element is the most recurrent in the final, since it is the core of breaking [1,24,28]. The rest of the categories remain neutral without abrupt changes between rounds, showing consistency in the basic structure of the confrontations.

Our results show the overall behavior of the rounds throughout the 2011–2021 period. We can see that the time of the round, toprocks, drops and powermoves decrease as the battle progresses, with the highest values in the first two rounds and small peaks in the final two rounds. This is because producing a strong finish is key to capturing the judges’ attention. The first and last rounds are therefore the most important so the dancer can start and finish in a forceful way [24]. Consequently, the powermove is most frequent in the first two and final two rounds. The toprock does not show strong variations compared to other categories. This could be due to the fact that, in the 30–40 s that the round lasts [1], this element provides volume, without demanding too much energy, allowing a few moments of active recovery before returning to the ground.

For the first two rounds we observed a higher frequency of elements with peaks in the final two. The sequentiality and frequency of the elements may be influenced by the opponent, since what one dancer does directly or indirectly affects the other. It should be noted that elements can adapt to the movements of the opponent [24,28,29]. Contrary to popular opinion, we found that acrobatics are rarely used in a round. Moreover, they are more frequent in initial rounds than in closing ones, perhaps owing to the fact that these movements require very high amounts of energy and concentration and there is a high risk of making mistakes that alter the development of the battle, as is the case with the powermove [1,14].

We found that in the final two rounds the elements have a longer duration than in the first two rounds, probably as a final attempt to make impact before the judges make their final decision [1].

Therefore, the first two rounds are the ones that last the longest in the battle. There seems to be a relationship between what the first dancer asks and what the second dancer answers, showing a type of “adaptation” to the battle. The temporal and sequential curve tends to be downward sloping in the confrontation. Factors such as fatigue, the dancer’s adaptation to his opponent, resource management or even possible strategies may be the causes of such a tendency [24].

Our results show a comparison between the rounds of the first competitive period (2011–2016). In the first two rounds, the toprock is longer in duration, peaking at 9 s in the fifth round. The same occurs for the powermove, with a duration of 5 s in its first two rounds, peaking at 5 s in the sixth. The total round time decreases from rounds 1 and 2, conserving its values until the end, as if it were a conversation and everyone wanted to talk at the same volume [1,24,29].

Except for rounds 3 and 4, a greater number of elements are performed in the answer than in the question, since going second in a round provides the dancer with previous information (the actions of the first dancer) so they are able to respond. Moreover, the answer concludes the battle and thus remains longer in the judge’s mind [1,24,25,29]. The highest spin frequency (2.18 at round 6) coincides with the peak powermove frequency of the last round (3.61), confirming that both categories are in sync and tend to go hand in hand [1,25,27].

The results tell us that the dancers in the second competitive period (2017–2021) have similar behavior to those of the first period, with the categories having a longer duration in the first two rounds, descending as the battle progresses, probably due to accumulated fatigue [14]. As in the first competitive period, toprocks and powermoves stand out. The only differences are in drops, transitions and spins. Specifically, the frequency of drops and transitions is higher in the first two rounds, with a peak in transitions in the fifth round. The time of a spin coincides in the first two rounds (1.47 s), which is not coincidental, as the answer tends to match the question [24]. On the one hand, these results could be indicative of which elements carry more weight in a battle and which elements tend to be repeated, modified or optimized, without losing sight of the context and the fundamental components of breaking [1,24,28,29].

The comparison of the rounds between both competitive periods (2011–2016 vs 2017–2021) shows significant differences that confirm an evolution in approaches to dancing throughout the last decade. In general, the second competitive period (2017–2021) has higher values, with an increase in the content that the athlete has to cover, demonstrating that greater effort is required as the years advance. Thus, below, we will discuss some aspects that stand out in the second competitive period. Firstly, the duration of all the rounds is longer. In the dancer’s second round, toprock time is longer, presumably because performing this element could help to achieve energy savings [1]. In rounds 2, 3 and 4, drop time is higher, which shows higher content and greater variety of execution. Footwork time is higher in rounds 1, 2, 3 and 4, which indicates that this element has been gaining relevance. The first round comprises more elements, because it is one of the most important rounds, in which the dancer seeks to raise the bar for the answer [24,29]. Toprock frequency is higher in rounds 1, 2, 5 and 6, and drop frequency is higher in the first two rounds, which indicates a change of direction towards more content, looking for changes in levels [25]. Footwork is more frequent in the first round, again indicating greater relevance. The duration of an element is higher in rounds 3, 4 and 6, and the duration of footwork is higher in round 3. Both tend to provide a round with more volume, footwork being the core of breaking. The latter increased in frequency in the second period, prioritized more from the dancer’s second round onwards [1,24]. Clearly, all these data show a tendency towards a greater elaboration of the rounds.

### Study limitations

We must take into account that there are factors within breaking that are very difficult or impossible to quantify and measure, such as those of a psychological nature or those that are not known and limit the study related to age, adrenaline, diet, supplementation, hours and method of training, hours of rest, nerves, quality of sleep and circadian hygiene, jetlag, reliability and training of judges, the athlete’s habits, injuries or operations, etc. [1,9,23,24,29–34].

These subjective factors limit the study as they cannot be analyzed and measured yet there is little doubt that they influence the execution or direction of the battle. Furthermore, it should be taken into consideration that the data were extracted from the Red Bull BC One championships, which means the results should not be extrapolated to the entire breaking scene, as each dancer has a way and style of executing the movements, deciding what to prioritize. At the same time, it would be important to analyze if there are changes in approaches to dancing between men and women.

### Practical applications

With the results of the study, we have developed a model of the temporal structure of the battle, taking into account the evolution of breaking over the last five years (see Table 8).

**Table 8:** Temporal structure “type” of men’s battles.

Round 1		Round 2		Round 3		Round 4		Round 5		Round 6	
(bboy 1)		(bboy 2)		(bboy 1)		(bboy 2)		(bboy 1)		(bboy 2)	
Cond	Time	Cond.	Time	Cond.	Time	Cond.	Time	Cond.	Time	Cond.	Time
TR1	7.58	AC1	1.33	BU1	2.59	TR1	6.51	TR1	7.35	BU1	2.7
BU1	2.68	TR1	7.23	TR1	7.44	D1	1.05	D1	1.13	TR1	6.36
D1	1.09	D1	1.09	D1	1.13	FW1	4.23	FW1	4.29	AC1	1.67
FW1	4.29	FW1	3.88	FW1	4.53	AC1	1.43	PM1	1.61	D1	1.22
T1	1.25	PM1	1.29	PM1	1.29	FW2	4.23	FR1	1.48	FW1	3.81
PM1	1.39	T1	1.24	FR1	1.34	FR1	1.4	FW2	4.29	FR1	1.6
FR1	1.24	FW2	3.88	T1	1.29	PM1	1.51	FR2	1.48	PM1	1.54
FW2	4.29	T2	1.24	AC1	1.8	FR2	1.4	T1	1.23	FW2	3.81
T2	1.25	PM2	1.29	PM2	1.29	S1	1.8	PM2	1.61	S1	1.97
AC1	1.8	FR1	1.29	FR2	1.34	PM2	1.51	S1	1.5	FW3	3.81
D2	1.09	S1	1.47	FW2	4.53	T1	1.33	PM3	1.61	PM2	1.54
FW3	4.29	FR2	1.29	T2	1.29	BU1	3.92	T2	1.23	T1	1.44
PM2	1.39	PM3	1.29	PM3	1.29			AC1	1.63	FR2	1.6
S1	1.47	FW3	3.88	S1	1.23			BU1	3.38		
PM3	1.39	BU1	2.71								
FR2	1.24										

## Conclusions

The average length of a breaking battle was 193 seconds, with rounds ranging from 29–37 s, thus concentrating a great deal of effort, explosiveness and wear and tear in short periods of time. Between rounds there is little rest time, which is equivalent to the opponent’s performance time.

The powermove is still a mainstay in breaking and usually accompanied by a spin. As rounds progress, footwork gains relevance. Acrobatics play a much less relevant role than expected and blowups experienced a slight increase. Freezes are key to finishing rounds and are usually preceded by a powermove.

The first and last round of each dancer are the most important with greater use of powermoves, acrobatics or spins. The frequency and effectiveness of footwork has increased over the last five years. We find peaks of intensity in the last round of each dancer, showing that greater effort is made in the final phase of the battle.

There has been an evolution in approaches to dancing and battling. Breaking is evolving towards a more and more athletic and demanding standpoint. The effort that the athlete must make is increasingly greater and will therefore require better preparation to cope with the exhausting demands of an Olympic future.

We propose a model of temporal structure of the battle through which breaking professionals will be able to develop more precise and adequate training.
